# Surfactants alter mosquito’s flight and physical condition

**DOI:** 10.1038/s41598-023-29455-6

**Published:** 2023-02-09

**Authors:** Aya Kato-Namba, Toshiaki Iida, Kazumi Ohta, Masahiro Suzuki, Kazuma Saito, Kohei Takeuchi, Maki Sakamoto, Hokto Kazama, Takao Nakagawa

**Affiliations:** 1 Personal Health Care Research, Kao Corporation, 2-1-3 Bunka, Sumida-ku, Tokyo, 131-8501 Japan; 2 RIKEN CBS-KAO Collaboration Center, 2-1 Hirosawa, Wako, Saitama 351-0198 Japan; 3 RIKEN Center for Brain Science, 2-1 Hirosawa, Wako, Saitama 351-0198 Japan; 4 Analytical Science Research, Kao Corporation, 2-1-3 Bunka, Sumida-ku, Tokyo, 131-8501 Japan; 5 Graduate School of Arts and Sciences, The University of Tokyo, 3-8-1 Komaba, Meguro-ku, Tokyo, 153-8902 Japan

**Keywords:** Animal behaviour, Chemistry, Infectious diseases

## Abstract

Mosquitoes carry lethal pathogens for humans and hundreds of thousands of people are killed by mosquito-borne diseases every year. Therefore, controlling mosquitoes is essential to protect the lives of people around the world. Insecticides are highly effective in controlling mosquitoes and have been used extensively worldwide. However, they have potentially harmful effects on biodiversity and environment, and some mosquitoes are resistant to insecticide ingredients and survive upon their application. Therefore, there is a demand for a method to control mosquitoes without using conventional insecticide ingredients. Here, we used *Aedes albopictus* to test whether solutions with low surface tension, particularly surfactant solutions can alter mosquito behavior by spreading over the hydrophobic cuticle of mosquitoes. We found that solutions with low surface tension indeed attached to mosquitoes flying or resting on the wall, and made them fall. In addition, solutions with yet lower surface tension covered the mosquito surface more quickly and widely, knocking down or killing mosquitoes. These results suggest that surfactants such as sodium dioctyl sulfosuccinate can be used to alter mosquito behavior without relying on conventional insecticides.

## Introduction

Mosquitoes transmit various vector-borne diseases such as dengue fever, malaria, Japanese encephalitis and Zika fever. Therefore, controlling mosquitoes is crucial to protect people from these diseases. *Aedes* mosquitoes, *Aedes aegypti*, and *Aedes albopictus*, are major vectors of dengue virus, chikungunya virus, yellow fever virus, and Zika virus, making these mosquitoes an important factor in the worldwide burden of infectious diseases^1^. Although vector control using insecticides is a conventional way to decrease transmission of these viruses to humans, it is threatened by the emergence of insecticide resistance^2^. Therefore, methods to control mosquitoes without using conventional insecticides are in need.

Mosquito body and wings are highly hydrophobic due to the numerous micro- and nano-scale structures on their surface, enabling droplets of water to roll off and the dirt to be removed from the body^3,4^. On the other hand, hydrophobic liquids such as polydimethylsiloxane can wet mosquito tarsi and induce an escape response upon tarsal contact^5^. Therefore, application (e.g. spraying) of hydrophobic solutions with low surface tension might prevent mosquitoes from flying or resting on the wall. If this were the case, mosquitoes can be controlled without the use of conventional insecticides.

Surfactants, surface-active agents, have both a hydrophilic and a hydrophobic group, which lower surface tension. Surfactants have been widely used in daily products and cosmetics and also commonly used in plant pesticides as additives to enhance the wetting and improve the biological efficacy of pesticide ingredients on plant leaves^6^. Moreover, surfactants can enhance wettability of the housefly surface and improve the distribution of aqueous pesticides by virtue of hydrophobic interaction that decreases solid–liquid interfacial tension^7^. However, the effects of the surfactant itself on mosquitoes remain unclear.

Here, we investigated whether aqueous solutions containing surfactants can alter mosquito behavior. We found that surfactant solutions with surface tension of about 30 mN/m can wet mosquitoes flying or resting on the wall and make them fall. Furthermore, solution with lower surface tension wetted the mosquito faster and wider, resulting in a decrease in mosquito activity and death. These results suggest that solutions with low surface tension can alter mosquito behavior without the use of conventional insecticides.

## Results

### Surfactant solutions with low surface tension can make mosquitoes fall

**Figure 1:**
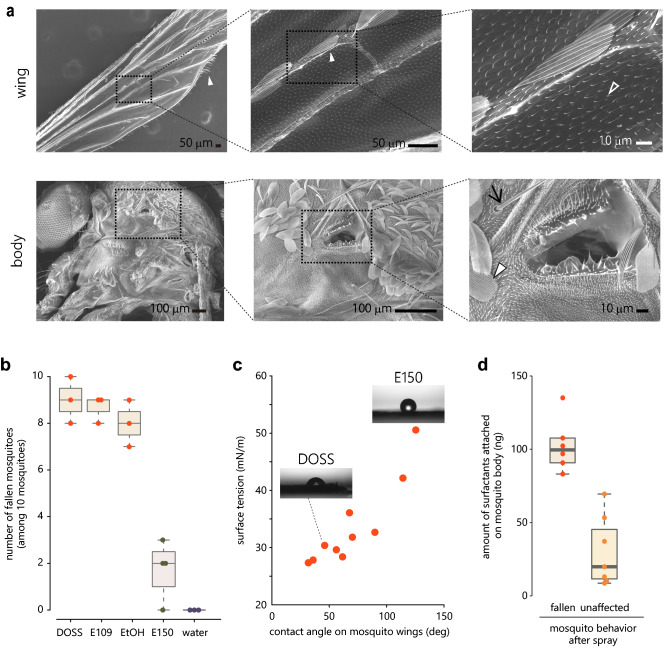
Solution with low surface tension can wet mosquitoes and make them fall. (**a**) SEM image of a surface of mosquito’s wings (top) and body (bottom). Mosquito body and wings were covered with scale (filled arrowhead), micro cilia (open arrowhead) and cilia (arrow). (**b**) The effect of each sprayed solution on mosquitoes staying on the wall. The number of mosquitoes (out of 10) that fell from the wall after spraying 3 mM DOSS, 3 mM E109, 70% EtOH, 3 mM E150, and pure water. (**c**) Correlation between the contact angle on mosquito wings and surface tension of surfactant solution. Droplets of E150 and DOSS on mosquito wings are shown in the pictures. Each dot indicates a surfactant. The surfactants used are DOSS, lauroylaminopropyldimethylamine oxide, alkyl benzyl dimethyl ammonium chlorides, polyoxyethylene (5) lauryl ether, polyoxyethylene (6) lauryl ether, E109, polyoxyethylene (12) lauryl ether, polyoxyethylene (19) lauryl ether, E150 and lauryl glucoside. The concentration for all the surfactant solutions is 3 mM. (**d**) The amount of DOSS attached on the body of fallen (n = 6) and unaffected mosquitoes (n = 7) after spraying 2 mM DOSS.

We first observed the surface of mosquito body and wings in detail using scanning electron microscopy. Scales were recognized on veins running throughout the wings as well as on the edges of wings (Fig. 1a, top-left, middle). Furthermore, micro cilia less than 10 μm in length covered the entire surface of the wings (Fig. 1a, top-right). The body of mosquitoes was also covered with fine scales and cilia approximately 100 μm in length (Fig. 1a, bottom-left). In addition, micro cilia less than 10 μm in length covered the entire body (Fig. 1a, bottom-middle, right). The edge of spiracles was also covered with cilia approximately 20 μm in length (Fig. 1a, bottom-right). These micro-scale structures likely underlie the hydrophobicity of the mosquito surface.

We hypothesized that solutions with low surface tension such as surfactant solutions can attach to these mosquito surfaces and consequently affect the behavior of the animals. To test this hypothesis, we applied surfactant solutions of anionic DOSS (sodium dioctyl sulfosuccinate, surface tension: 28 mN/m in 3 mM) and nonionic E109 (polyoxyethylene (9) lauryl ether, surface tension: 32 mN/m in 3 mM), and 99% ethanol (22 mN/m) to mosquitoes. We also tested 3 mM nonionic E150 (polyoxyethylene (47) lauryl ether) as a surfactant solution with higher surface tension (51 mN/m), and pure water (surface tension: 72 mN/m). We found that spraying some of these solutions from a distance of 30 cm make mosquitoes on the mesh wall fall on the ground. Solutions with low surface tension, DOSS, E109 and ethanol made most mosquitoes fall (Fig. 1b and Supplementary Video S1 online, DOSS: 9.3 ± 0.8, E109: 8.7 ± 0.6, EtOH: 8.0 ± 1.0 out of 10 mosquitoes fell). On the other hand, E150 solution made only 4.4 ± 3.5 mosquitoes fall, and water had no effect at all (Fig. 1b and Supplementary Video S2 online). These results show that solutions with low surface tension can make mosquitoes fall from the wall. The fact that E150 and water had little effect also indicates that the mere wind and liquid flows caused by spraying are not sufficient to make mosquitoes fall.

To quantify the relationship between surface tension and the ability to wet mosquito body, we measured static surface tension and the contact angle on mosquito’s wings of various surfactant solutions, and found a positive correlation between the two (Fig. 1c, adjusted R-squared = 0.80). This suggests that surfactant solution with lower surface tension can more efficiently wet the mosquito body, and thus make more mosquitoes fall.

To examine how much surfactant is necessary to make mosquitoes fall, we quantified the amount of surfactant molecules remaining on the mosquito body after spraying the surfactant solution. To collect sufficient numbers of both fallen and unaffected mosquitoes, here we sprayed 2 mM DOSS solution to flying mosquitoes from a distance of 40 cm instead of spraying 3 mM DOSS solution from a distance of 30 cm that made most of the mosquitoes fall (Fig. 1b), and collected six fallen and seven unaffected mosquitoes. LC/MS analysis revealed that more surfactant molecules were attached on fallen than unaffected mosquitoes with 112.5 ± 36.3 ng of DOSS attached on each fallen mosquito body (Fig. 1d, unaffected: 30.3 ± 23.8 ng). Therefore, on average, 112.5 ng of surfactant molecules is necessary to make mosquitoes fall.

### Surfactants affect mosquito flight

**Figure 2:**
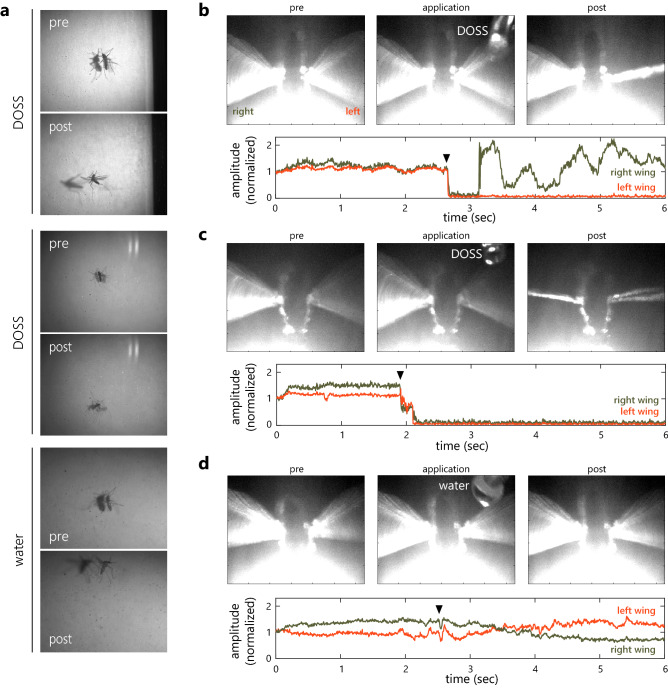
Surfactants affect mosquito flight. (**a**) Snapshots of movies taken with a high-speed camera capturing the behavior of mosquitoes on the wall after being sprayed with DOSS (top and middle) or water (bottom). Mosquitoes fall and fly upward in response to DOSS and water, respectively. (**b**–**d**) (Top) Mosquito flight behavior before (pre), during (application), and after (post) the application of 3 mM DOSS (**b**,**c**) or water (**d**) on the left wing. (Bottom) Wing beat amplitude of the left and the right wings recorded with microphones set in the immediate vicinity of the wing tips. Filled arrowhead indicates the timing of application of solution.

To further investigate the immediate behavioral effect of surfactants, we observed mosquitoes with a high-speed camera. When water was sprayed on mosquitoes resting on a piece of paper, they responded promptly by flying upward (Fig. 2a and Supplementary Video S3 online). On the other hand, when surfactant solution was sprayed, mosquitoes fell down (Fig. 2a and Supplementary Videos S4 and S5 online). To examine the change in mosquito flight behavior in more detail, we applied a small amount of surfactant solutions (100–150 nL) or water (800–1000 nL) to the left wing of a tethered mosquito flying in a flight simulator, and analyzed the change in the wing beat amplitude. After the application of DOSS, mosquitoes became unable to move their left wing or both wings (Fig. 2b,c). In contrast, the flight was not affected by the application of water (Fig. 2d). These results demonstrate that attachment of surfactant solution on mosquito wings prevents wing movement and flight, resulting in the falling behavior.

### Solutions with further lower surface tension can knock down or even kill mosquitoes

**Figure 3:**
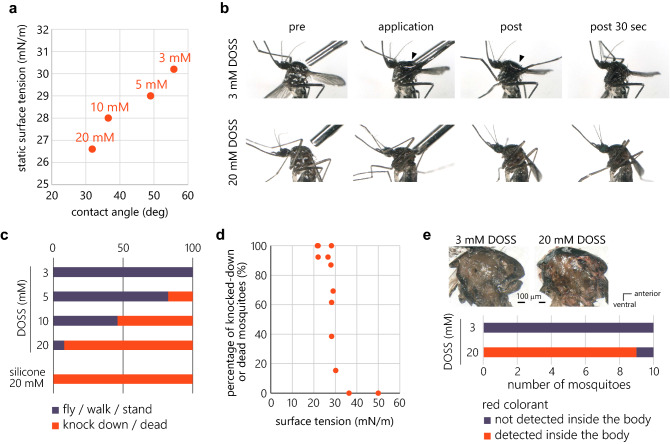
Solutions with lower surface tension can knock down or even kill mosquitoes. (**a**) The relationship between the contact angle on mosquito body and surface tension of 3, 5, 10, 20 mM DOSS. (**b**) Snapshots of movies showing wetting behavior of DOSS solutions on mosquitoes. Each photo shows solutions and mosquitoes before (pre), during (application), immediately after (post) and 30 s after the application of 3 and 20 mM DOSS (post 30 s). Droplets were observable only when 3 mM DOSS was applied (filled arrowheads). Application of 20 mM DOSS did not form a drop as the solution spread rapidly over the surface. (**c**) Mosquito behavior after the application of 3, 5, 10, 20 mM DOSS and silicone. 130 nL of each solution was applied on the mosquito’s thorax using a microliter syringe. Blue represents the percentage of mosquitoes that were able to fly, walk or stand 60 min after the application of each solution, and red represents the percentage of mosquitoes that were knocked down or dead. (**d**) Correlation between surface tension of solutions and the percentage of knocked-down or dead mosquitoes. Each dot indicates one of the following solutions; 3, 5, 10, 20 mM DOSS, 5, 10, 20 mM E108, 5, 10, 20 mM silicone, 20 mM E150, and 20 mM alkyl benzyl dimethyl ammonium chlorides. (**e**) Entry of 20 mM DOSS solution into the mosquito’s body. Red colorant was detected inside the body following the application of 20 mM but not 3 mM DOSS containing red colorant on the mosquito’s thorax.

We wondered if solutions with yet lower surface tension would exert stronger behavioral impacts on mosquitoes. We confirmed that both static surface tension and the contact angle decreased as the concentration of DOSS is increased (Fig. 3a). A drop of 20 mM DOSS solution applied to the thorax spread quickly over the mosquito surface, while a drop of 3 mM DOSS solution remained on the mosquito surface for some time before it spread over the surface, indicating that 20 mM DOSS can wet the mosquito body more rapidly (Fig. 3b, Supplementary Video S6 (20 mM DOSS) and S7 (3 mM DOSS)). A longer-term effect of DOSS was analyzed by observing the mosquitoes 60 min after its application and classifying the behavioral state into either flying, walking, standing, knocked-down (mosquitoes are flat on back, but can move their legs) or dead (immobile upon mechanical stimulation). We found that the number of knocked-down or dead mosquitoes increased dose-dependently (Fig. 3c). To investigate whether this phenomenon is DOSS- or surface tension-dependent, we similarly applied silicone oil (Siloxanes and Silicones, 3-hydroxypropyl Me, ethers with polyethylene glycol mono-Me ether), which has static surface tension lower than that of 20 mM DOSS. Silicone oil was as effective as 20 mM DOSS, suggesting that surface tension is the determinant of this phenomenon (Fig. 3c).

Analysis of the relationship between surface tension of various solutions and percentage of knocked-down or dead mosquitoes revealed that the behavioral effect of solutions dramatically increases below the surface tension of 30 mN/m (Fig. 3d).

Finally, we found that the solution with lower surface tension goes into the mosquito body. We applied 3 mM and 20 mM DOSS solution containing a red colorant to the mosquito thorax and cut the body through the sagittal section 60 min after the application. Red colorant was detected in all the tested mosquitoes following the application of 20 mM DOSS solution whereas it was detected in only 1 out of 10 mosquitoes following the application of 3 mM DOSS solution (Fig. 3e). These results suggest that solutions with low surface tension knock down or kill mosquitoes by rapidly covering the surface of mosquito body and further suffocate mosquitoes by entering the body via spiracles.

## Discussion

Mosquitoes seek hosts and find mates in flight. Therefore, it is essential for mosquitoes to maintain accurate flight and posture in air. Compared to other flying insects, mosquito wingbeat kinematics show high wingbeat frequency, low wingbeat amplitude, and large, rapid spanwise rotations^8^. Here, we found that surfactant solutions with low surface tension make mosquitoes fall. This is likely caused by two factors. First is the direct prevention of wing movement by efficiently wetting the wings. On a rough surface like mosquito body and wings, several patterns of wetting phenomena are observed depending on contact angle on the surface^9^. When a droplet of surfactant solution is attached to the mosquito body, it spreads and covers the mosquito. As shown in Supplementary Videos S6 and S7 online, 20 mM DOSS more rapidly covered the mosquito body than 3 mM DOSS, suggesting that solutions with lower contact angle and surface tension can wet mosquitoes more efficiently. Second is the intervention of the function of sensilla on the wings. Because there are multiple somatosensory and gustatory sensilla on insect wings^10^ that monitor insect’s posture during flight^11^, the surfactant solution may have also altered flight by inhibiting the function of these sensilla.

We found that solutions with yet lower surface tension can further knock down or kill mosquitoes. What could be the mechanism underlying this stronger effect on mosquitoes? The respiratory system of insects consists of branching tubes, the tracheae, that open to the exterior through valve-like “spiracles”. Tracheae ramify throughout the body and end in minute tubules, the tracheoles, where gaseous exchange between air and tissues occurs^12^. In *Drosophila melanogaster*, which belongs to the *Diptera* as with mosquitoes, there are two and seven pairs of spiracles in the thorax and the abdomen, respectively^13^. Insect spiracles are essential for oxygen uptake as well as carbon dioxide release, and a unidirectional air flow from anterior towards posterior spiracles have been reported in several insects^14^. In fact, a study reported that flies die within approximately 10 min after all the 18 spiracles are completely blocked^15^. Furthermore, certain mineral oil kills insects including lice by physically blocking the spiracles^16,17^. In this study, we found that mosquitoes are knocked down or killed by 20 mM DOSS solution, which enters the mosquito body after the application. These lines of evidence suggest that surfactant solutions with low surface tension rapidly covers the surface of mosquitoes before evaporating, blocks the entire spiracles, and inhibits gas exchange, which lead to death. Because silicone with low surface tension was as competent as 20 mM DOSS in inducing the behavioral effect, surface tension likely is the determinant of this phenomenon. To further reveal the impact of surfactant solutions, it is necessary to measure various aspects of mosquito’s physiology including respiration rate, metabolism and gas exchange after application. For example, Leis et al. measured respiratory patterns and metabolism in *Rhodnius prolixus* using a syringe-type respirometric chamber^18^.

In summary, we revealed that surfactant solutions well attached to mosquitoes flying or resting on the wall, and made them fall down. In the natural environment, mosquitoes can be preyed on by ants and spiders after they fall, resulting in the decrease in the number of mosquitoes. Furthermore, solutions with lower surface tension wet mosquito’s body more widely and quickly, knocking down or killing mosquitoes. In practice, we envision spraying these solutions using sprayers and sprinklers. We therefore propose that application of surfactant solutions is an effective measure in controlling mosquitoes. This method has a potential to overcome the problem of insecticide resistance and control mosquitoes potently as well as safely. In the future, it is important to understand the mechanism through which surfactant solutions affect the physical condition of mosquitoes. Moreover, it is necessary to assess the effect of surfactants applied in the field by following the number of mosquitoes and the patients with mosquito-borne diseases over time.

## Methods

### Reagents

Reagents shown in Figs. 1 and 3 were purchased from or offered by the following companies. DOSS (sodium dioctyl sulfosuccinate, CAS No. 577-11-7): Tokyo Chemical Insdustry Co., Ltd./polyoxyethylene (5) lauryl ether (CAS No. 9002-92-0), E108 (polyoxyethylene (6) lauryl ether, CAS No. 9002-92-0), E109 (polyoxyethylene (9) lauryl ether, CAS No. 9002-92-0), polyoxyethylene (12) lauryl ether (CAS No. 9002-92-0), polyoxyethylene (19) lauryl ether (CAS No. 9002-92-0), E150 (polyoxyethylene (47) lauryl ether, CAS No. 9002-92-0), lauryl glucoside (CAS No. 110615-47-9), alkyl benzyl dimethyl ammonium chlorides (CAS No. 68424-85-1), sodium lauryl sulfate (CAS No. 151-21-3): Kao Corporation/lauroylaminopropyldimethylamine oxide (CAS No. 7732-18-5): Kawaken Fine Chemicals Co., Ltd./ethanol (CAS No. 64-17-5): FUJIFILM Wako Chemicals Corporation/silicone (siloxanes and silicones, 3-hydroxypropyl methyl, ethers with polyethylene glycol monomethyl ether, CAS No. 117272-76-1): Shin-etsu chemical Co., Ltd.

Surface tension of each solution was measured using the Wilhelmy plate method using a platinum plate at 25 °C (Tensiometer K100, KRÜSS Optic GmbH).

### Mosquito rearing and maintenance

Female mosquitoes *Aedes albopictus* were reared at 28 °C, 70% relative humidity with a photoperiod of 12 h light:12 h dark (lights on at 8 AM). Eggs of *Aedes albopictus* were purchased from Sumika Technoservice Corporation and allowed to hatch in deoxygenated, deionized water. Larvae were fed TetraMin Baby (Tetra). Pupae were placed in a small cup of deionized water and moved to a 30 cm × 30 cm × 30 cm-insect cage (BugDorm-1, Mega View Science Co., Ltd), and allowed to eclose. Adult mosquitoes were mated in the insect cage for at least 1 week. Mosquitoes were provided with unlimited access to 10 wt% sucrose (Wako chemicals) solution. Experiments were performed during 10 am–5 pm using mosquitoes 14–21 days after eclosion.

### SEM analysis

Mosquitoes were killed by freezing and fixed on a holder with dental wax (GC utility wax, GC corporation). The surface structure of mosquitoes was determined by a scanning electron microscope (SEM) (JSM-IT500, JEOL).

### Analysis of the effect of sprayed solutions on mosquitoes

10 mosquitoes were put in a custom-made white acryl box (6 cm × 6 cm × 4 cm), whose front and back side were covered with mesh cloth. We sprayed each solution with a trigger sprayer (Yoshino Corporation) to mosquitoes resting on the mesh cloth at a distance of 30 cm and counted the number of fallen mosquitoes while taking a movie (4K Video Camera HC-WX2M, Panasonic).

### Contact angle measurement

For the experiment shown in Fig. 1c, female mosquitoes were killed by placing them in the fridge and wings were removed with tweezers. A total of 10 wings were placed on double-sided tape cut to 15 mm × 15 mm (No. 5000NS, Nitto Denko Corporation), which was fixed on a glass slide (S2441, Matsunami Glass Ind., Ltd.). For the experiment shown in Fig. 3d,e, female mosquitoes were killed by placing them in the fridge and legs and wings were removed with tweezers. Mosquito scales were collected by rolling a mosquito with tweezers on double-sided tape cut to 15 mm × 15 mm, which was fixed on a glass slide. This procedure was repeated 10 times until mosquito scale covered the surface of the tape. Droplets (0.5 μL) of solutions were deposited on the mosquito samples, and the contact angle was measured after 1 s (Fig. 1c) and 60 s (Fig. 3d,e) using a contact angle meter (Drop Master, Kyowa Interface Science Co., Ltd).

### LC/MS analysis

To assess the amount of surfactant molecules on fallen and unaffected mosquitoes after spraying, we sprayed 2 mM DOSS solution to mosquitoes flying in the mosquito net at a distance of 40 cm and collected 7 unaffected and 6 fallen mosquitoes. The mosquitoes were stored individually in screw-top glass vials (Maruemu Corporation) in the fridge until extraction. We added 1 mL of 50% methanol (Kanto chemical Co., Inc.) solution to the vials and extracted DOSS on mosquito body by vigorously vortexing them for 1 min. DOSS on mosquito body was quantified by high-performance liquid chromatography coupled to mass spectroscopy using LCMS2020 (Mass spectrometer; Shimadzu). The injection volume was 5 μL for each sample solution. L-column2 ODS (3 μm, 2.1 × 150 mm, Chemicals evaluation and research institute) was used as the analysis column. The HPLC solvent consisted of solvent A (50% methanol containing 100 mM ammonium acetate) and solvent B (95% methanol containing 100 mM ammonium acetate). The samples were separated with a linear gradient from 50 to 100% solvent B with a low rate of 300 nL/min using HPLC. The mass spectrometer was operated in a selected ion monitoring (SIM) mode with *m/z* 421.20 for DOSS. Calibration lines were generated by plotting the concentration against the peak area of DOSS.

### High-speed camera recording of mosquito behavior in response to sprayed solutions

Five mosquitoes were placed and acclimated for 5 min in a custom-made transparent acryl tube (diameter: 150 mm, height: 420 mm). A piece of white paper was vertically positioned in the middle of the tube on which the mosquitoes were allowed to stay. DOSS solution or water was sprayed from the top of the tube and the behavioral responses of mosquitoes were monitored using a high-speed camera (Mini UX, Photon Ltd.) with a shutter speed of 2000 fps.

### Analysis of mosquito behavior in a flight simulator

For experiments using a flight simulator shown in Fig. 2b,c, at most 10 pupae of *Aedes albopictus* were kept in a 5 mL-cup (Neo Mini cup No.5, Maruemu Corp.) placed within a polypropylene bottle (170 mL, AS-115, Thermofisher), and allowed to eclose. Adult male and female mosquitoes were kept in the same bottle to allow them to mate freely. We confirmed that female mosquitoes raised in this condition can be activated by exhaled air as those raised in cages. Experiments using a flight simulator were performed using mosquitoes 5 days after eclosion.

Individual mosquitoes were cold anesthetized, removed of their legs, tethered to a stainless steel pin, and transferred to the previously reported flight simulator composed of a semi-circular array of green LEDs^19^. The setup was enclosed in an opaque container and the mosquito’s flight was recorded using an infrared camera. The sound of the left and the right wing beat (wing beat amplitude) was monitored using two microphones, each of which was positioned laterally ~ 1 mm away from the tip of the extended wing on each side of the mosquito. The visual stimuli were vertical gratings with 60 deg^−1^ spatial frequency. 100–150 μL of surfactants or 800–1000 μL of water was applied to the left wing of a flying mosquito with a microliter syringe (86250, Hamilton) and analyzed the change in wing beat amplitude.

### Analysis of a spread of solutions applied on the mosquito thorax

Individual mosquitoes were anesthetized on ice and subsequently kept on a Peltier plate (CP-085, Scinics) set at 5 °C. 130 nL of solution was applied on the center of the mosquito thorax with a microliter syringe (86250, Hamilton). The mosquito was then transferred into a clear plastic cup (200 mL, 39811, Nipro) and observed for 60 min by taking a movie (4K Video Camera HC-WX2M, Panasonic). The cup was gently shaken by tapping 2–3 times with a finger 60 min after the application of each solution and the mosquitoes were examined whether they could fly, walk, stand, were knocked down or dead. If mosquitoes were flat on back but could move their legs, the mosquitoes were considered as “knocked down”. If mosquitoes were completely immobile, they were considered to be dead. In the experiments shown in Fig. 3b, a movie was taken at 30 fps and snapshots were extracted with ImageJ^20^.

For experiments shown in Fig. 3e, mosquitoes were anesthetized on ice and 250 μL of 3 or 20 mM DOSS containing 0.25% red colorant (Red No. 504, Ponceau SX) was applied on the thorax. After 60 min of application, mosquitoes were frozen and cut through the sagittal plane with a double edge blade (FA-10, thickness: 0.1 mm, FEATHER safety razor Co., Ltd.). The presence of red colorant inside the thorax was examined under a digital microscope (VHX-6000, Keyence corporation).

## Supplementary Information

Supplementary Information.

Supplementary Video S1.

Supplementary Video S2.

Supplementary Video S3.

Supplementary Video S4.

Supplementary Video S5.

Supplementary Video S6.

Supplementary Video S7.

## Data Availability

The datasets used and/or analysed during the current study are available from the corresponding author on reasonable request.

## References

[1] McGraw EA, O'Neill SL (2013). Beyond insecticides: New thinking on an ancient problem. Nat. Rev. Microbiol..

[2] Moyes CL (2017). Contemporary status of insecticide resistance in the major *Aedes* vectors of arboviruses infecting humans. PLoS Negl. Trop. Dis..

[3] Matthews BJ, Younger MA, Vosshall LB (2019). The ion channel ppk301 controls freshwater egg-laying in the mosquito *Aedes aegypti*. Elife.

[4] Byun D (2009). Wetting characteristics of insect wing surfaces. J. Bionic Eng..

[5] Iikura H (2020). Mosquito repellence induced by tarsal contact with hydrophobic liquids. Sci. Rep..

[6] Lin H, Zhou H, Xu L, Zhu H, Huang H (2016). Effect of surfactant concentration on the spreading properties of pesticide droplets on Eucalyptus leaves. Biosyst. Eng..

[7] Wan Q (2020). The wetting behavior of three different types of aqueous surfactant solutions on housefly (*Musca domestica*) surfaces. Pest Manag. Sci..

[8] Bomphrey RJ, Nakata T, Phillips N, Walker SM (2017). Smart wing rotation and trailing-edge vortices enable high frequency mosquito flight. Nature.

[9] Spori DM (2008). Beyond the lotus effect: Roughness influences on wetting over a wide surface-energy range. Langmuir.

[10] Tsubouchi A (2017). Topological and modality-specific representation of somatosensory information in the fly brain. Science.

[11] Ai H (2013). Sensors and sensory processing for airborne vibrations in silk moths and honeybees. Sensors (Basel).

[12] Demerec, M. *Biology of drosophila*. Facsimile ed edn, (Cold Spring Harbor Laboratory Press, 1994).

[13] Lehmann FO (2001). Matching spiracle opening to metabolic need during flight in Drosophila. Science.

[14] Heinrich EC, McHenry MJ, Bradley TJ (2013). Coordinated ventilation and spiracle activity produce unidirectional airflow in the hissing cockroach, *Gromphadorhina portentosa*. J. Exp. Biol..

[15] Heymann N, Lehmann FO (2006). The significance of spiracle conductance and spatial arrangement for flight muscle function and aerodynamic performance in flying Drosophila. J. Exp. Biol..

[16] Wolf L, Eertmans F, Wolf D, Rossel B, Adriaens E (2016). Efficacy and safety of a mineral oil-based head lice shampoo: A randomized, controlled, investigator-blinded, comparative study. PLoS One.

[17] Taverner P, Beattie A (2002). Modes of action against arthropods, Ch 2. Spray Oils Beyond 2000: Sustainable Pest and Disease Management.

[18] Leis M, Pereira MH, Casas J, Menu F, Lazzari CR (2016). Haematophagy is costly: Respiratory patterns and metabolism during feeding in *Rhodnius*
*prolixus*. J. Exp. Biol..

[19] Badel L, Ohta K, Tsuchimoto Y, Kazama H (2016). Decoding of context-dependent olfactory behavior in Drosophila. Neuron.

[20] Schneider CA, Rasband WS, Eliceiri KW (2012). NIH Image to ImageJ: 25 years of image analysis. Nat. Methods.

